# The effect of different intracanal irrigants on the push-out bond strength of dentin in damaged anterior primary teeth

**DOI:** 10.25122/jml-2024-0164

**Published:** 2024-05

**Authors:** Leila Bassir, Shirin Taravati, Farzad Nouri, Saeide Rahimi

**Affiliations:** 1 Department of Pediatric Dentistry, School of Dentistry, Ahvaz Jundishapur University of Medical Sciences, Ahvaz, Iran; 2 Department of Oral and Maxillofacial Surgery, School of Dentistry, Zahedan University of Medical Sciences, Zahedan, Iran

**Keywords:** anterior, primary teeth, push-out bond, canal, irrigation

## Abstract

This experimental study investigated the effect of different intracanal irrigants on the push-out bond strength of dentin in damaged anterior primary teeth. The crowns of 90 anterior primary teeth were sectioned horizontally, 1 mm above the cementoenamel junction (CEJ). Following canal preparation with K-files, all groups except the negative control received normal saline irrigation. Canals were then irrigated with either 3% or 5.25% sodium hypochlorite (NaOCl), 2% or 0.2% chlorhexidine (CHX) solution (except negative and positive controls). The roots were filled with Metapex material and covered with a calcium hydroxide liner. In root canals, the bond was applied by self-etching and then light-cured for 20 seconds before canals were restored incrementally with composite. Stereomicroscopes were used to assess failure patterns. Push-out bond strengths (MPa ± SD) were: 3% NaOCl (16.92 ± 5.78), 5.25% NaOCl (8.96 ± 3.55), 2% CHX (14.76 ± 5.56), and 0.2% CHX (7.76 ± 2.93). Significant differences were seen across the irrigants regarding the push-out bond strength of dentin sections (P <0.001). The most frequent failures were adhesive and cohesive. NaOCl and CHX irrigants increased the push-out bond strength compared to controls. Compared to controls, both 3% NaOCl and 2% CHX irrigants significantly increased the push-out bond strength of dentin in non-vital anterior primary teeth.

## INTRODUCTION

Preserving primary teeth is crucial for their role in the development and future eruption of permanent teeth. Premature loss of anterior milk teeth can lead to numerous complications, including decreased chewing efficiency, loss of vertical dimension of occlusion, parafunctional habits, speech problems, malocclusion, and delays in the development of permanent teeth [1-7]. Early Childhood Caries often cause premature loss of primary teeth, potentially resulting in malocclusion, incorrect tongue positioning, and speech disorders [8]. The treatment process can be challenging and disruptive for children and their parents, who may need to adjust work schedules for multiple dental visits [9].

Dental decay is a major reason for primary tooth loss, and pulp therapy is a treatment option to prevent tooth loss. For primary teeth with irreversible pulpitis or necrosis, root canal treatment (RCT) is required, which involves mechanical cleaning, irrigation, and placement of resorbable fillings [10]. The choice of irrigation solution in RCT is critical due to its antimicrobial and tissue-dissolving properties. However, some solutions can weaken the bond strength of adhesive systems to root dentin, alter structural components like collagen, and affect mechanical properties such as bending strength and elastic modulus.

Chlorhexidine gluconate (CHX) 2% is commonly used for root canal cleanings due to its antimicrobial properties, long-lasting effect, and low toxicity. CHX rinse solution also helps maintain the bond strength between resin material and root dentin by preventing the collapse of collagen fibers [8,11-14]. Normal saline is another widely used irrigation solution for children because it has no side effects and can be used alongside other irrigants. Sodium hypochlorite (NaOCl) is frequently used for broad applications in dental root canal preparation [15-24].

In endodontic infections, preventing the root canal from becoming infected is crucial to the success of the root canal treatment, particularly during root resorption or first tooth formation [25]. There is a need to investigate the effect of varying irrigation solutions with different concentrations on the bonding of dental pulp chambers and root canals, especially concerning the bond strength to dentin. Effective canal cleaning solutions are essential for disinfecting canals and preparing dentin surfaces for optimal bonding with composite materials. Methods such as micro shear, micro tensile, and push-out tests are used to evaluate bond strength to dentin walls, with the push-out test yielding results more aligned with clinical conditions due to its ability to replicate fractures parallel to the dentin-bond interface [26-33].

Additionally, certain intracanal coatings have been recommended to reduce bacterial presence. Treatments for traumatized teeth, periapical lesions, revascularization of immature teeth, apexification, and inflammatory root resorption often utilize intracanal filling materials. Metapex, a material containing calcium hydroxide, iodoform, and silicone oil medium, is commonly used due to its superior antimicrobial effects compared to simple calcium hydroxide [34-39].

In the present investigation, we examined the impact of different root canal rinses on the push-out bond strength of Metapex-filled cervical dentin in the roots of anterior primary teeth. Understanding these effects is crucial for optimizing dental treatment strategies in pediatric dentistry, ensuring durable restorations and long-term oral health for young patients.

## MATERIAL AND METHODS

### Sample preparation and treatment

After crown cutting and pulp tissue harvesting, anterior deciduous (milk) teeth were selected for inclusion in the study. Following root canal preparation and cleaning with either NaOCl (at concentrations of 3% and 5.25%) or CHX (at concentrations of 2% and 0.2%), Metapex filler material was applied. Additionally, G-Premio bonding and Z250 composite repair were utilized, with the teeth being cut perpendicular to the longitudinal axis prior to these applications. The bond strength values were measured in megapascals (MPa) using an Instron bond strength test machine. Forces were applied until the moment of failure to determine the bond strength. To calculate the sample size, Cochran's formula was used in the research (N: sample size, α: first type error, 1-β: power of the test, d: margin of error, S: standard deviation):


$$N=\frac{{\left(z_{1-\alpha /2}+z_{1-\beta }\right)}^{2}*s^{2}}{d^{2}}\frac{{\left(z_{1-\alpha /2}+z_{1-\beta }\right)}^{2}*s^{2}}{d^{2}}$$

### Research implementation method

**Figure 1:**
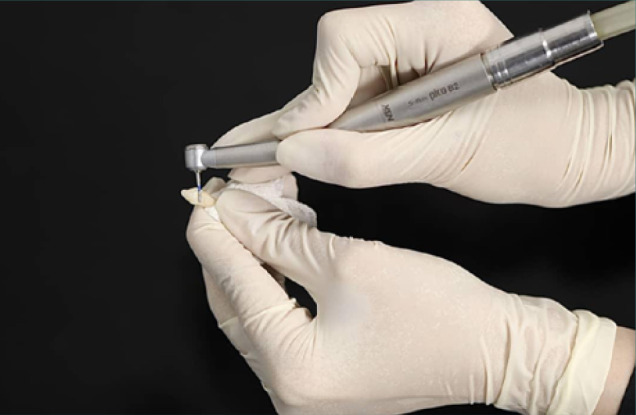
Removing the tooth crown horizontally with a diamond bur

Ninety extracted anterior deciduous teeth were evaluated. At least two-thirds of the root and one-third of the crown length were required for inclusion. The teeth were randomly divided into six groups: Group 1: 3% NaOCl, Group 2: 5.25%NaOCl, Group 3: 2% CHX, Group 4: 0.2% CHX, Group 5: positive control (Normal saline), and Group 6: Negative control. Teeth were immersed in 0.5% chloramine T solution and stored at 4°C for one week before experiments began. Crowns were cut horizontally 1 mm above the cementoenamel junction (CEJ) with a diamond bur (Teezkavan), and the apex was sealed with a calcium hydroxide liner (Figure 1).

### Root canal preparation

A 10 ml saline solution was used to wash the root canals of the teeth after the pulp tissue was removed. Root canals were prepared using a K manual file up to number 45 and a piezo reamer size 2 with a diameter of 0.8 mm to shape uniform, parallel spaces. The roots of each group (except the positive control) were washed with 5 ml of the assigned washing solution during the same period: NaOCl 3%, NaOCl 5.25% (Hyponic 5.25%, Nikdarman), CHX 2% (Clorex 2%, Nikdarman). Solutions were prepared by diluting 5.25% NaOCl and 2% CHX with normal saline to obtain 3% NaOCl and 0.2% CHX.

### Filling and bonding

**Figure 2:**
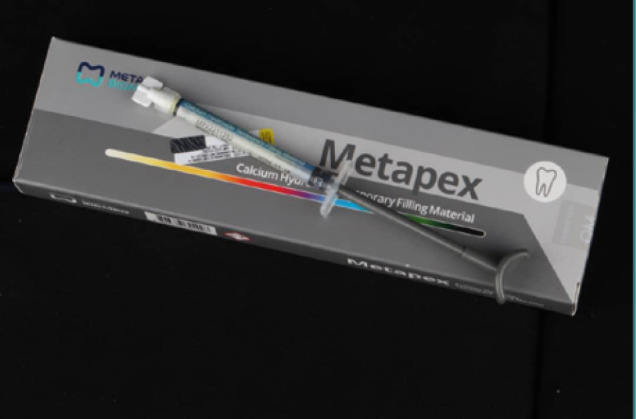
Metapex root canal filling material

Standardized roots were dried using a paper cone (Dentsply) number 40 and filled with Metapex material (Metapex, Metadental) using a special syringe head and packing technique (Figure 2) (following the manufacturer's instructions) and covered with a calcium hydroxide liner with a thickness of 1 mm (Bisco, TheraCal).

**Figure 3:**
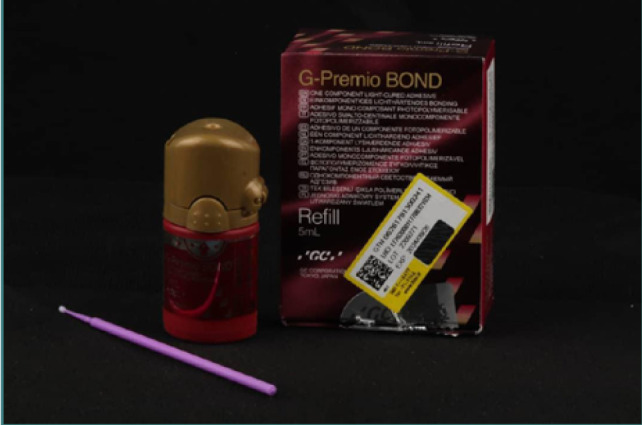
G-Premio generation 8 bonding factor

A short composite post was reinforced using G-Premio eighth-generation bonding (GC, G-Premio bond) with the self-etch method. The bond was applied inside the root canal using a microbrush (Microbrush X, Microbrush, Grafton) in two layers. After application, the layers were dried for 5 seconds with high airflow and then cured with an LED light (Bluephase C5, Ivoclar Vivadent Clinical) for 20 seconds (Figure 3).

**Figure 4:**
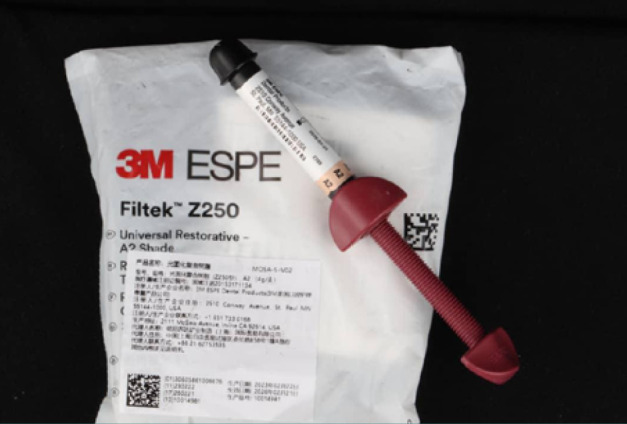
Z250 composite

Each layer of the Z250 composite (3M ESPE) was applied in 2 mm increments using the incremental technique (Figure 4) and cured for 40 seconds. The canal was filled up to the CEJ. The samples were stored in a laboratory oven (Universal Oven, Memmert GmbH) at 100% humidity and 37°C for one week. The teeth were mounted in a cold-curing acrylic resin block (Caulk, DENSPLY Maillefer). Abrasive cutting machines were then used to cut the roots perpendicular to the longitudinal axis, producing three to four slices of 1 mm thickness from each root.

Forces were applied to samples using a cylindrical stainless-steel plunger with different diameters (0.8, 1, and 1.2 mm) according to the channel size. The samples were mounted on an Instron device to measure the push-out bond strength. Push-out forces were applied at a cross-sectional speed of 0.5 mm/min until bond failure occurred. Bond strength was calculated in megapascals by dividing the force in newtons (N) by the bonding area. The bonded area was calculated using the 2πr×h relationship. In this regard, π equals 3.14, r is the radius of the intra-radicular space, and h is the height in mm.

Samples were examined under a stereomicroscope at 30x magnification to identify the type of failure.

- *Adhesive failure* occurs when the filling material completely separates from the dentin, leaving the dentin surface free of any filling material.
- *Cohesive failure* occurs within the filling material or dentin itself. It is classified as cohesive in composite if the failure occurs within the filling material and cohesive in dentin if it occurs within the dentin. The dentin surface remains covered with filling material.
- *Mixed failure*: this is a combination of adhesive and cohesive failure patterns, where the filling material covers part of the dentin surface while other parts are exposed.

### Statistical analysis

Data were analyzed using SPSS 0.25 software. The mean and standard deviation of the push-out bond strength were calculated for each group. ANOVA was used to compare bond strength values, with pairwise comparisons conducted using the least significant differences (LSD) test. Chi-square tests were used to compare the frequency of failure patterns. A significance level of 0.05 was used for all statistical tests.

## RESULTS

### Descriptive findings

**Figure 5:**
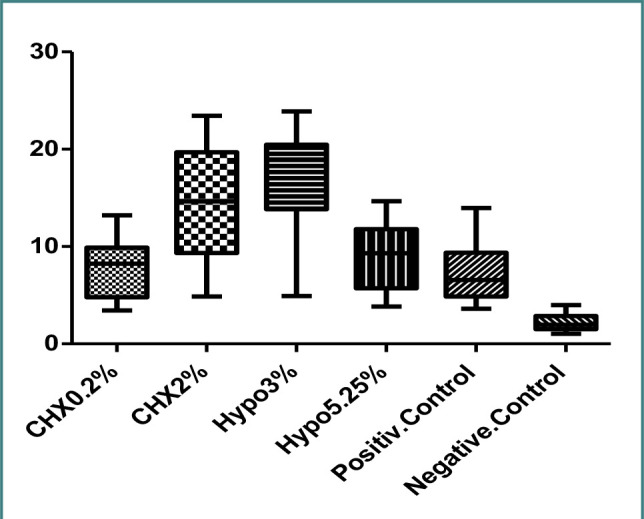
. The mean and 95% confidence interval of the mean push-out bond strength of dentin in anterior primary teeth washed with different solutions

An experimental laboratory study was conducted on 90 anterior primary teeth. The root canals of the teeth were washed with four different solutions: 3% NaOCl, 5.25% NaOCl, 0.2% CHX, and 2% CHX. Results were compared with positive and negative control groups. The mean ± standard deviation of the push-out bond strength of dental slices was 16.92 ± 5.78 MPa for NaOCl 3%, 8.96 ± 3.55 MPa for NaOCl 5.25%, 14.76 ± 5.56 MPa for CHX 2%, and 7.76 ± 2.93 MPa for CHX 0.2%. In the positive control group, the push-out bond strength was 7.13 ± 3.06 MPa, while in the negative control group, it was 2.22 ± 0.86 MPa (Table 1 and Figure 5).

**Table 1:** Mean, standard deviation, minimum, and maximum push-out bond strength values (MPa)

Max	Min	Deviation	Mean	Group
23.90	4.91	5.78	16.92	NaOCl 3%
14.66	3.84	3.55	8.96	NaOCl 5.25%
23.43	4.86	5.56	14.76	CHX 2%
13.22	3.43	2.93	7.76	CHX 0.2%
13.97	3.61	3.06	7.13	Positive control
3.99	1.05	0.86	2.22	Negative control

### Analytical findings

The Kolmogorov-Smirnov and Shapiro-Wilk test was used to confirm the data followed a normal distribution (*P* >0.05). Statistical analyses were therefore conducted using parametric tests. A one-way analysis of variance (ANOVA) revealed significant differences in push-out bond strength between the groups (*P* <0.001). The negative control group had the lowest average bond strength values (2.22 MPa), while the highest values were observed in the 3% NaOCl group (16.92 MPa). Comparisons using the LSD test revealed several significant differences. The CHX group showed a significantly higher average bond strength than the negative control group by 2% (*P* < 0.001, mean difference = -12.54). The average bond strength of the 3% NaOCl group was significantly higher than the negative control group (*P* < 0.001, mean difference = -14.69). Similarly, the 5.25% NaOCl group had significantly higher average bond strength compared to the negative control group (*P* < 0.001). The positive control group also showed significantly higher average bond strength compared to the negative control group (*P* = 0.001, mean difference = -4.91). No significant difference was found between the positive control and 0.2% CHX group. However, the 2% CHX group had significantly higher average bond strength than the positive control group (*P* < 0.001, mean difference = -7.62). Significant differences were also observed between the positive control and 3% NaOCl groups, with the latter having higher average bond strength (*P* < 0.001, mean difference = 9.78). There was no significant difference between the positive control and 5.25% NaOCl groups in terms of average push-out bond strength.

In this study, the average push-out bond strength did not significantly differ between the 5.25% NaOCl and 0.2% CHX groups. However, there were significant differences between the 5.25% NaOCl and 2% CHX groups. The 2% CHX group had a higher average bond strength compared to the 5.25% NaOCl group (*P* = 0.001, mean difference = -5.79). Additionally, substantial differences were found between the 3% NaOCl and 5.25% NaOCl groups, with the 3% NaOCl group showing higher average bond strength (*P* = 0.001, mean difference = -7.95). Significant differences were observed when comparing the 0.2% CHX and 3% NaOCl groups, with the 3% NaOCl group having higher average bond strength (*P* = 0.001, mean difference = 9.16). However, there were no significant differences in the average push-out bond strength between the 3% NaOCl and 2% CHX groups.

Finally, the average bond strength values between the 2% CHX and 0.2% CHX groups were significantly different, with the 2% CHX group having higher values (*P* < 0.001, mean difference = 7.01) (Table 2).

**Table 2:** Comparison of push-out bond strength among different canal washing solutions

Group 1	Group 2	Mean difference	*P* value
Negative control	Positive control	-4.91	0.001
Negative control	CHX 0.2%	-5.53	<0.001
Negative control	CHX 2%	-12.54	<0.001
Negative control	NaOCl 5.25%	-6.74	<0.001
Negative control	NaOCl 3%	-14.69	<0.001
Positive control	CHX 0.2%	-0.621	0.67
Positive control	CHX 2%	-7.62	<0.001
Positive control	NaOCl 5.25%	-1.83	0.213
Positive control	NaOCl 3%	-9.78	<0.001
CHX 0.2%	CHX 2%	7.01	<0.001
CHX 0.2%	NaOCl 5.25%	1.20	0.410
CHX 0.2%	NaOCl 3%	9.16	<0.001
CHX 2%	NaOCl 5.25%	-5.79	<0.001
CHX 2%	NaOCl 3%	2.15	0.143
NaOCl 5.25%	NaOCl 3%	-7.95	<0.001

The fracture patterns observed in the different treatment groups are summarized in Table 3. In the 3% NaOCl group, there were two cases (13.2%) of adhesive fracture, five cases (33.4%) of cohesive fracture in dentine, five cases (33.4%) of cohesive fracture in composite, and three cases (0.20%) of mixed fracture. The 5.25% NaOCl group had four cases (26.7%) of adhesive fracture, five cases (33.3%) of cohesive fracture in dentin, two cases (13.3%) of cohesive fracture in composite, and four cases (26.7%) of mixed fracture. In the 2% CHX group, there were three cases (0.20%) of adhesive fracture, four cases (26.7%) of cohesive fracture in dentin, five cases (32.4%) of cohesive fracture in composite and three cases (0.20 %) of mixed fracture. In the 0.2% CHX group, there were 4 cases (26.7%) of adhesive fracture, 4 cases (26.7%) of cohesive fracture in dentine, four cases (26.7%) of cohesive fracture in composite and three cases (20.0%) of mixed fracture. In the negative control group, all the fractures were of adhesive type. Still, in the positive control group, there were twelve cases (0.80%) of adhesive-type fractures and three cases (0.20%) of cohesive fractures. Based on the chi-square test results, significant differences were found between the groups in terms of failure frequency (*P* <0.001).

**Table 3:** The frequency and percentage of different fracture patterns in damaged anterior milk teeth after washing with different irrigants

Group	Adhesive (%)	Cohesive dentin (%)	Cohesive composite (%)	Mixed (%)	Total (%)
NaOCl 3%	2 (2/13)	5 (4/33)	5 (4/33)	3 (20)	15 (0/100)
NaOCl 5.25%	4 (7/26)	5 (4/33)	2 (4/13)	4 (7/26)	15 (0/100)
CHX 2%	3 (20)	4 (7/26)	5 (4/32)	3 (20)	15 (0/100)
CHX 2/0 %	4 (7/26)	4 (7/26)	4 (7/26)	3 (20)	15 (0/100)
Positive control	12 (0/80)	0	3 (0/20)	0	15 (0/100)
Negative control	15 (0/100)	0	0	0	15 (0/100)

## DISCUSSION

This study found that the push-out bond strength of anterior primary teeth was increased by NaOCl and CHX irrigants compared to control groups. The NaOCl 3% and CHX 2% groups showed the strongest bond strength values. Various factors, such as the type of tooth, the degree of mineralization of dentin, the amount of dentin bonded surfaces, the type of bond strength testing method, environmental humidity, and test conditions, affect bond strength in laboratory conditions. However, acid pH, solvent type (water, ethanol, or acetone), and filler percentage also affect bond strength values, and these variables have caused bond strength values to vary widely in existing studies [40-46]. The dentin far from the pulp is more calcified, and because the tubules are different in diameter in other areas of the tooth, they have a higher bond strength [40]. Morphological differences in dentine affect bond strength. In order to maintain the function and maintain the tooth until it can be permanently replaced, pulp-involved milk teeth should be restored. One of the challenges in composite restorations is the strength of a composite bond to the enamel or dentin of milk teeth following endodontic or restorative treatments. The present study was conducted to determine whether root canal cleaners affect the push-out bond strength values of dentin roots of damaged anterior deciduous teeth.

This research found that the strongest push-out bonds were obtained when the roots of the damaged anterior milk teeth were washed with 3% NaOCl (mean 16.92 MPa), 2% CHX (mean 14.76 MPa), 5.25% NaOCl (mean 8.96 MPa) and 0.2%, CHX (mean 7.76 MPa), respectively. In all comparisons, the bond strength values of different irrigant groups differed significantly from those of the negative control group. A significant difference was observed between the positive control group and the CHX 2% and NaOCl 3% groups. In terms of the experimental groups, the difference between NaOCl 3% and CHX 0.2% was significant, as was the difference between CHX 2% and CHX 0.2%; 5.5% NaOCl and 3% NaOCl, 5.25 NaOCl, and 2% CHX. However, there was a significant difference in push-out bond strength between 5.25% NaOCl and 0.2% CHX irrigants. There were no differences between the groups treated with 3% NaOCl and 2% CHX. The dentin samples from the 0.2% CHX and 5.25% NaOCl groups had lower bond strength values than those in the 3% NaOCl and 2% CHX groups.

According to Meshki *et al*. [47], the push-out bond strength of 5^th^, 6^th^, 7^th^, and 8^th^ generation bonding agents was determined after composite posts were applied to anterior milk teeth. The study was conducted in a laboratory setting with 60 extracted anterior milk teeth that still had at least two-thirds of their root length. In total-etch and self-etch patterns, the bond strength of the 8^th^-generation bonding agent was significantly higher than the 5^th^, 6^th^, and 7^th^-generation bonds. The bond strength of the 5^th^, 6^th^, and 7^th^ generation bonding agents did not differ significantly. Additionally, the bond strength of the 8^th^-generation agents was higher in self-etch patterns compared to total-etch patterns, suggesting that 8^th^-generation bonding agents are effective for bonding composite posts to dentin in anterior primary teeth [47].

In the present study, the push-out bond strength was relatively higher when using a 3% sodium hypochlorite washing solution compared to 2% CHX and 0.2% CHX. Sodium hypochlorite is the most common root canal washing solution [31,48-51] due to its excellent properties, including the ability to dissolve organic content and necrotic tissue in the smear layer [48]. Fibryanto *et al*. [49] investigated the effects of washing canals with 5.25% and 2.5% NaOCl on total-etch adhesive bond strength to dentin following the application of nano-filled resin composite and found that 5.25% NaOCl yielded the highest bond strength values. However, our study recorded the highest bond strength values for dentin samples in the 3% NaOCl group [49].

The increase in dentin bond strength due to NaOCl is attributed to its deproteinization activity. NaOCl dissolves and removes encapsulated dentin collagen after acid etching, creating mineralized dentin surfaces for adhesive resin application. This process allows adhesive resin to connect directly to dentin without a collagen-reinforced resin layer, known as the hybrid layer [50,51]. However, some researchers argue that NaOCl's removal of collagen fibrils from the dentin surface and prevention of a permanent hybrid layer formation may weaken the bond between adhesive systems and dentin walls, as collagen significantly influences adhesive adhesion. Uniformity of dentin collagen, particularly after using root canal cleaning agents, is a critical factor in bonding failure within the root canal system [52-54]. In the study of Fibryanto *et al*. [49], washing the root canal system with 5.25% NaOCl and 2.5% NaOCl caused a decrease in dentin collagen. In a recent study, despite the weakest collagen staining in the 5.25% NaOCl group, the highest values of shear bond strength were observed, indicating that collagen may not improve the initial adhesion in bonding, which needs further investigation.

The dentin of baby teeth has a high organic content and a lower mineral content than that of permanent teeth. These differences may reduce the strength of the dentin-resin bond in this substrate and increase the possibility of degradation over time [55]. Therefore, the preventive effects of matrix metalloproteinase inhibitors may be more pronounced in primary teeth than in permanent teeth.

In the present study, the push-out bond strength values of anterior primary teeth dentin after washing with CHX at both concentrations were lower than those obtained with sodium hypochlorite. These differences were especially significant compared to the 3% sodium hypochlorite concentration. On the other hand, the push-out bond strength values of dentin samples washed with 2% CHX were approximately double those of samples washed with 0.2% CHX. CHX is a cationic biguanide with optimal antimicrobial activity at a pH of 5.5 to 7.0. Its antimicrobial function works by adsorption onto the cell walls of microorganisms and disruption of their intracellular components. Several researchers have investigated the effects of pretreatment with CHX on dentin bond strength, with mixed results regarding whether CHX decreases immediate bond strength [56,57]. Some studies suggest that CHX adsorption by dentin may improve resin penetration into the dentin, although this requires further evaluation. The binding of CHX to loose and surface apatites interferes with the performance of primer monomers. Additionally, dentin contains matrix metalloproteinases (MMPs), activated by self-etch and total-etch adhesives. Their activity in the hybrid layer destroys type I collagen and reduces the durability of the bond [58].

In laboratory conditions, CHX can inactivate all matrix metalloproteinases in dentin at concentrations of 0.02% [59]. CHX has high substantivity, and this chemical property of CHX in situ conditions is due to non-specific binding related to its positive charge, making its effects last longer than the application period. Therefore, the concentration of CHX might have a limited impact in this context [52,55,60]. However, in this study, the bond strength values of dentin samples washed with 2% CHX were significantly higher than those washed with 0.2% CHX. This discrepancy may be related to the specific conditions of the current research, including the type of bonding agent and the etching pattern used.

The present study used Metapex filling material to fill root canals. Research indicated that Metapex can reduce the bond strength of composite to dentin. This reduction is due to Metapex residues, which affect bonding by dissolving under exposure to adhesive primers containing ethanol and acetone [61]. G-Premio eighth-generation bonding was utilized in this study using the self-etch method to prepare root canals in anterior primary teeth. Eighth-generation bonding agents can be applied using either the total-etch or self-etch methods. It has been reported that the bond strength to dentin in self-etch systems is higher due to less microleakage and prevention of excessive drying after etching. For this reason, the self-etch method is preferred to increase bond strength and reduce microleakage in dentin. Additionally, self-etch systems are favored for dentin due to its greater sensitivity to decalcification caused by less mineralization and more tubules. Conversely, total-etch systems are preferred for enamel because they remove the smear layer more effectively, although they may leave a gap between the dentin and the monomer [62].

This research showed that most failures in the washing or positive control groups were either adhesive or cohesive failures in the dentin and composite. Also, in the positive control group (normal saline), 80% of the failures were adhesive, and in the negative control group, all the failures were adhesive. In the control groups, no cases of mixed failures were seen, and in general, the least failures were of mixed type. The difference in the frequency of failure patterns in different research is due to the inconsistency and non-uniformity of the composition and particle size of the materials, which affects their penetration inside the dentinal tubules [63].

This research used the push-out method to estimate the bond strength to the dentin of anterior primary teeth. This test applies shear forces to the interface between the composite and the dentin, simulating clinical conditions more accurately than the linear shear method [64]. This research used a cylindrical stainless-steel plunger according to the channel size with dimensions of 0.8 mm, 1 mm, and 1.2 mm. One limitation of the current research is that it used only two washing solutions and a single root canal-filling material. Including a greater variety of washing solutions and canal-filling materials could provide more comprehensive results. Additionally, the research samples were not subjected to long-term aging or thermal cycling, which could influence the outcomes. Despite efforts to standardize all parameters in this study, some unavoidable differences in the shape and size of the roots existed.

## CONCLUSION

Irrigant solutions significantly increased the push-out bond strength of anterior primary teeth compared to the control groups. The highest bond strength values were observed in the groups treated with 3% sodium hypochlorite and 2% CHX.

## Data Availability

Further data is available from the corresponding author upon reasonable request.
